# ERK inhibition represses gefitinib resistance in non-small cell lung cancer cells

**DOI:** 10.18632/oncotarget.24147

**Published:** 2018-01-10

**Authors:** Mengfan Qi, Ye Tian, Wang Li, Dan Li, Tian Zhao, Yuxin Yang, Qiwen Li, Sujun Chen, Yan Yang, Zhixiong Zhang, Liang Tang, Zhonghua Liu, Bo Su, Fei Li, Yonghong Feng, Ke Fei, Peng Zhang, Fan Zhang, Lei Zhang

**Affiliations:** ^1^ Shanghai Key Lab of Tuberculosis, Shanghai Pulmonary Hospital, Tongji University School of Medicine, Shanghai 200433, China; ^2^ Department of Thoracic Surgery, Shanghai Pulmonary Hospital, Tongji University School of Medicine, Shanghai 200433, China; ^3^ Clinical Translational Research Center, Shanghai Pulmonary Hospital, Tongji University School of Medicine, Shanghai 200433, China; ^4^ The Central Laboratory, Shanghai Pulmonary Hospital, Tongji University School of Medicine, Shanghai 200433, China; ^5^ School of Life Science and Technology, Tongji University, Shanghai 200092, China; ^6^ Department of Biology, New York University, New York, NY 10003, USA

**Keywords:** non-small cell lung cancer, gefitinib resistance, ERK signaling, autophagy

## Abstract

Gefitinib, an EGFR tyrosine kinase inhibitor, is used to treat non-small cell lung cancer (NSCLC) patients with activating EGFR mutations. However, the resistance to gefitinib eventually emerges in most of the patients. To understand its mechanism, we generated two acquired gefitinib-resistant NSCLC cell lines. The resistant cells have slower growth rates, but are more resistant to apoptosis in the presence of gefitinib, compared with their sensitive counterparts. In addition, our genome-wide transcriptome analysis reveals unexpected pathways, particularly autophagy, are dysregulated in the gefitinib-resistant cells. Autophagy is significantly enhanced in resistant cells. Importantly, inhibition of autophagy reduces gefitinib resistance. Furthermore, the phosphorylation of ERK, the extracellular signal-regulated kinase, is activated in resistant cells. Inhibition of ERK phosphorylation abrogates gefitinib resistance by suppressing autophagy both *in vitro* and *in vivo*. These findings establish a link between ERK and autophagy in gefitinib resistance, and suggest that the ERK signaling may serve as the potentially therapeutic target for treating gefitinib resistance in NSCLC patients.

## INTRODUCTION

Tyrosine kinase inhibitors (TKIs), such as gefitinib and erlotinib, have been used as the first-line treatment for non-small cell lung cancer (NSCLC) patients harboring oncogenic alterations in epidermal growth factor receptor (EGFR) [1–4]. TKIs act as competitive inhibitors for ATP binding of EGFR, thus blocking cell proliferation signaling pathways, leading to apoptosis of cancer cells [5]. Although the initial clinical response and antitumor activity are encouraging, most patients eventually develop acquired resistance towards these drugs, and die due to cancer metastasis from these resistant tumor cells. Therefore, TKI resistance presents a serious challenge in NSCLC treatment.

Multiple mechanisms of acquired resistance to EGFR inhibitors have been reported, including a secondary “gatekeeper” mutation in EGFR (T790M) [6], MET receptor gene amplification [7], the activation of TGF-β, NF-κB, or IGFIR signaling [8–10], or the pathological transformation of NSCLC to small cell lung cancer (SCLC), etc [11–12]. However, it has been reported that unknown mechanisms might also play a role in many cases of gefitinib resistance [11].

Autophagy is induced in drug-treated tumor cells [13–17]. Autophagy is an evolutionarily conserved process that is involved in the degradation and turnover of cytoplasmic proteins and organelles in response to environmental stress. During autophagy, a double-membraned vesicle called an autophagosome is formed, which then fuses with a lysosome to form an autolysosome. The enclosed proteins and organelles are degraded by the lysosomal enzymes and recycled to provide energy for the cells to survive. In this process, autophagy-related gene (Atg) products, such as the Atg5-Atg12-Atg16 complex and the LC3-II (Atg8-II) complex, are recruited to form the autophagosome [18, 19]. LC3 (Atg8) is cleaved and conjugated to phosphatidylethanolamine (PE) to form the membrane-bound, lipidated LC3-II, with the help of Atg7 and Atg3 [20]. LC3-II also binds to adapter proteins, including NBR1 and SQSTM1/p62, which are involved in trafficking proteins for autophagic degradation of ubiquitinated protein aggregates [21]. SQSTM1/p62 is normally degraded during autophagy and accumulates when autophagy is impaired [22]. Autophagy has a dual role in oncogenesis. In early tumorigenesis, autophagy may serves as a tumor suppression mechanism by degrading damaged organelles and limiting cell growth [23, 24]. However, in Ras-driven cancers, autophagy promotes cell survival, maintains oxidative metabolism and promotes tumorigenesis [25]. How autophagy is regulated during gefitinib resistance in NSCLC cells is not well understood.

Besides autophagy, the extracellular signal-regulated kinase (ERK) signaling pathway also plays a role in drug resistance. ERK is activated via a pathway that involves GTP-loading of Ras and the sequential phosphorylation and activation of Raf, MEKs, and ERK [26]. ERK activation is observed in multiple cancer cells that are resistant to MEK [27] or EGFR kinase inhibitors [28]. The Raf/MEK/ERK pathway can govern drug resistance, apoptosis and the sensitivity to the targeted therapy [29].

In this study, we generated two stable gefitinib-resistant NSCLC cell lines, and found that many unexpected cellular pathways are dysregulated in these cells. In particular, autophagy was induced in gefitinib-resistant cells. Inhibition of autophagy reduced the growth of resistant cells. Furthermore, resistant cells exhibited persistent ERK and AKT activation. Importantly, we showed, for the first time, that the inhibition of ERK greatly suppressed autophagy-induced gefitinib resistance both *in vitro* and *in vivo*. Therefore, our study establishes the novel link between the ERK signaling and autophagy in gefitinib resistance, and suggests the ERK signaling may serve as the potential therapeutic target for the treatment of gefitinib resistance in NSCLC patients.

## RESULTS

### Characterization of gefitinib-resistant NSCLC cells

To establish gefitinib-resistant cell lines, we subcutaneously injected the gefitinib-sensitive cells PC9 and HCC827 into the armpit of mice (Supplementary Figure 1A). When the xenograft tumor became visible 4 weeks later, 3 µM gefitinib was intraperitoneally administered to the mice twice per week. The tumor size was measured and recorded every week (Supplementary Figure 1B). At week 10, the gefitinib-resistant tumor was isolated, dissected into smaller pieces, and grown in culture dishes for 4 more weeks to establish the gefitinib-resistant NSCLC cell lines: PC9/GR and HCC827/GR (Supplementary Figure 1C).

**Figure 1:**
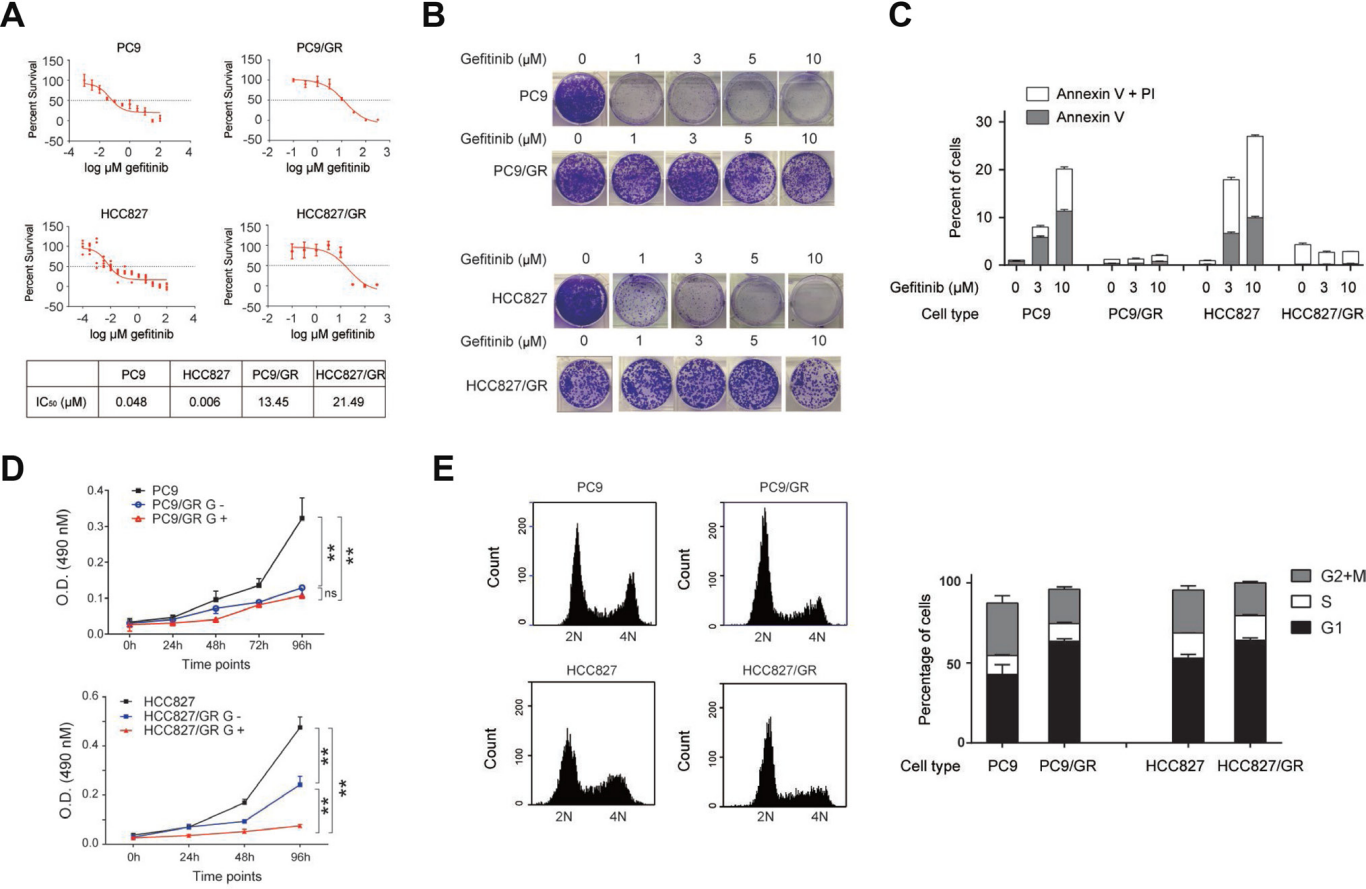
Characterization of gefitinib-resistant NSCLC cell lines (**A**) Survival of gefitinib-sensitive (PC9 and HCC827) and gefitinib-resistant (PC9/GR and HCC827/GR) cells in response to different concentrations of gefitinib. IC_50_ was given in the table (lower panel). (**B**) Colony formation assay of PC9, PC9/GR, HCC827, and HCC827/GR cells cultured in 0, 1, 3, 5, and 10 µM of gefitinib-containing media. (**C**) Flow cytometry analysis of apoptosis in PC9, PC9/GR, HCC827, and HCC827/GR cells, treated with 0, 3, and 10 µM gefitinib for 2 days. The bar graph represents percentages of cells expressing early (Annexin V) or late (PI) apoptosis markers. (**D**) Cell proliferation assay of PC9, PC9/GR, HCC827, and HCC827/GR cells measured at 0, 24, 48, 72, and 96 hours. G+ and G- represent the culture media with and without 3 µM gefitinib, respectively. (**E**) PC9, PC9/GR, HCC827, and HCC827/GR cells were subjected for flow cytometry based cell cycle analysis. The histograms show the representative results generated by Accuri C6 software (left panel). The bar graphs show the percentages of cells within each cycle phase (G1, S, G2, and M) for each cell type (right panel). Analysis was performed in triplicates. Data are represented as mean (SD).

To determine the IC_50_ of gefitinib for these cells, we cultured these cells with different concentrations of gefitinib, and carried out cell survival assays. We found there were significant difference in IC_50_ of gefitinib between gefitinib-sensitive and–resistant cells: 0.048 µM for PC9, 0.048 µM for HCC827 cells, 13.45 µM for PC9/GR cells, and 21.49 µM for HCC827/GR cells (Figure 1A).

Next, these cells were assessed for their colony formation ability in the presence of increasing concentrations of gefitinib (0, 1, 3, 5, 10 µM). We found that both PC9/GR and HCC827/GR cells had a stronger colony formation ability compared with their gefitinib-sensitive counterparts, PC9and HCC827 cells (Figure 1B). Most of the sensitive cells had no colony formation in the presence of 1 µM gefitinib, but most of the resistant cells were able to form colonies in the presence of 10 µM gefitinib. This observation demonstrates the clonogenic outgrowth capacity of the resistant cells is much stronger than their sensitive counterparts.

To understand whether this discrepancy is due to their resistance to apoptosis, we performed flow cytometry assays using the early and late apoptosis markers, Annexin V and PI, respectively, to detect the extent of apoptosis. Our data showed that, in the presence of 0, 3, and 10 µM of gefitinib for 2 days, 1%, 8%, and 20% of PC9 cells, and 1%, 18%, and 26% of HCC827 cells, became apoptotic (Figure 1C), indicating that gefitinib treatment induces apoptosis in the gefitinib-sensitive cells. However, the percentage of apoptotic cells didn’t increase for the resistant cells at all, which remained at approximately 1% for PC9/GR cells and 5% or less for HCC827/GR cells, for increasing amounts of gefitinib treatments. These results indicate that gefitinib-resistant cells are resistant to apoptosis under the gefitinib treatment.

To compare the proliferation rate between the gefitinib-sensitive and -resistant cells, we performed cell proliferation assay, and found that PC9/GR and HCC827/GR cells grew considerably slower in the presence of 3 µM gefitinib (labeled as G+) than PC9 and HCC827 cells. Upon withdrawing the gefitinib treatment (labeled as G-), HCC827/GR cells were able to recover the proliferation rate more rapidly than the PC9/GR cells (Figure 1D). To understand whether the slow proliferation rate was due to changes in cell cycle progression, we found that the percentage of cells in G1 phase increased from 43% in PC9 cells to 63% in PC9/GR cells, but those in G2 and M phases decreased from 33% in PC9 cells to 21% in PC9/GR cells in average (Figure 1E). Similarly, the percentage of cells in G1 phase increased from 53% in HCC827 cells to 64% in HCC827/GR cells, but those in G2 and M phases decreased from 27% in HCC827 cells to 20% in HCC827/GR cells in average (Figure 1E).

These data indicate that PC9/GR and HCC827/GR cells have much higher IC_50_ of gefitinib than their sensitive counterparts, which is consistent with their stronger anti-apoptotic ability. In addition, the slow proliferation rate of PC9/GR and HCC827/GR cells was due to their increased presence in G1 phase and decreased presence in G2 and M phases during the progression of the cell cycle.

### Transcriptome analysis of gefitinib-resistant cells

**Figure 2:**
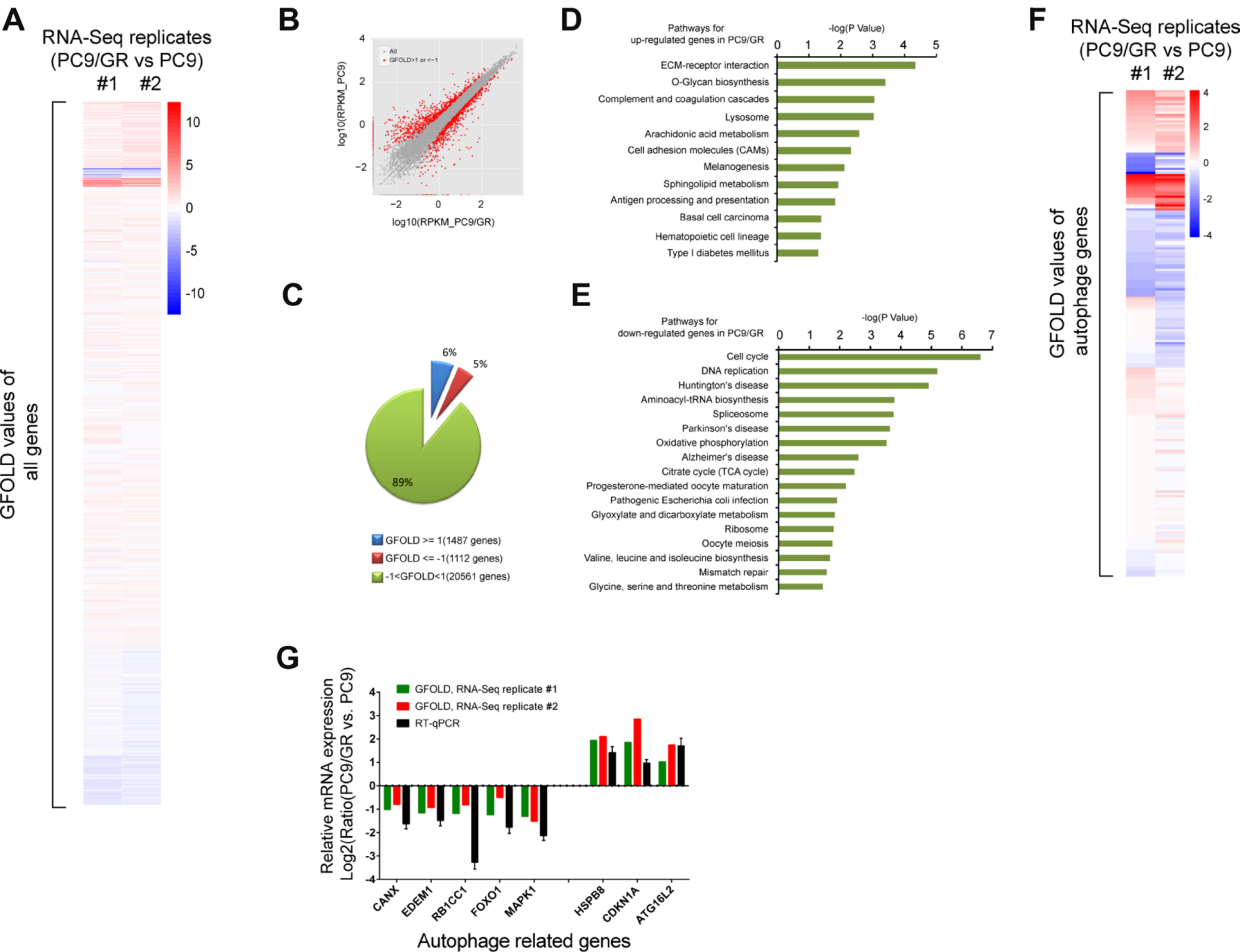
Transcriptome analysis of gefitinib-resistant cells (**A**) Heatmap clustering of all genes based on GFOLD values from independent mRNA-Seq replicates #1 and #2. Red: up-regulated genes in PC9/GR cells compared to those in PC9 cells. Blue: down-regulated genes in PC9/GR cells compared to those in PC9 cells. (**B**) Comparison of the differentially expressed genes in the PC9/GR (X axis) and PC9 cells (Y-axis), as measured by the GFOLD values. The grey dots represent all genes, the red dots represent those genes in the PC9/GR cells with GFOLD values of either >1 or <-1 compared with PC9 cells; (**C**) 6% of the total detected genes (1487 genes) were up-regulated (GFOLD >= 1) and 5% of the total detected genes (1112genes) were down-regulated (GFOLD <= –1) in the PC9/GR cells. The remaining 89% genes of the total detected genes have GFOLD values between -1 and 1. (**D**–**E**) KEGG pathways that were significantly enriched for the up- (D) or down-regulated (E) genes in PC9/GR cells compared with the PC9 cells using DAVID analysis (*P* <= 0.05). (**F**) Heatmap clustering of autophagy-related genes based on GFOLD values. Blue: down-regulated genes in PC9/GR cells compared to those in PC9 cells. (**G**) Correlation of mRNA expression from mRNA-Seq and RT-qPCR for selected autophagy genes. Y axis represents the Log2 transformed mRNA expression levels from three experiments: mRNA-Seq replicate #1 and #2, and RT-qPCR.

To determine what pathways are dysregulated in gefitinib-resistant cells, we generated two sets of mRNA-Seq data to compare the transcriptomes of PC9 and PC9/GR cells using two independent biological replicates. Gene expression was quantified in rpkm (reads per kilobase of exon per million mapped sequence reads) (Supplementary Table 3). Next, GFOLD was used to rank the differentially expressed genes from our RNA-Seq data. The GFOLD value can be considered as a reliable log2-fold change [30]. The heatmap clustering analysis showed that GFOLD values of most genes between these two biological replicates correlate very well in terms of differentially expressed genes (Figure 2A). In addition, hundreds of genes were either down- or up-regulated in PC9/GR cells compared with PC9 cells (Figure 2B). Genes with GFOLD values >1 or <-1 are labeled as red dots. Specifically, 6% of total detected genes (1487 genes) were up-regulated in PC9/GR cells, with a GFOLD value over 1, and 5% of total detected genes (1112 genes) were down-regulated, with a GFOLD value less than -1, in one replicated experiment (Figure 2C).

Next, we performed KEGG pathway enrichment analysis for the top 2000 down- or up-regulated genes in PC9/GR cells using DAVID (Supplementary Table 4). The KEGG pathways that were significantly (*P* <= 0.05) enriched for up-regulated genes included ECM-receptor interaction, O-Glycan biosynthesis, lysosome, cell adhesion molecules (CAMs) (Figure 2D). By contrast, the KEGG pathways that were significantly enriched for down-regulated genes included cell cycle, DNA replication, oxidative phosphorylation, the citrate cycle (TCA cycle), and ribosome (Figure 2E).

Since lysosome activity is closely related to autophagy, we carried out heatmap clustering analysis of autophagy related genes, and the results showed that autophagy related genes have very similar expression patterns in both replicated experiments (Figure 2F). Among 232 autophagy related genes, based on GFOLD values, we chose three most up-regulated genes: HSPB8 [31], CDKN1A [32], and ATG16L2 [33], which are known to positively regulate autophagy, and five most down-regulated genes: CANX [34], EDEM1 [35], RB1CC1 [36], FOXO1 [37], and MAPK1 [38], which are known to be involved in the regulation of autophagy, for validation by RT-qPCR. We found that the log2 ratio of normalized gene expression in PC9/GR vs. those in PC9 cells from our RT-qPCR results were consistent with the GFOLD values from two replicates of mRNA-Seq data (Figure 2G).

In conclusion, our mRNA-Seq analysis reveals multiple pathways involved in gefitinib-resistant NSCLC cells, and importantly, identified key genes dysregulated in the autophagy pathway enhanced in PC9/GR cells.

### Autophagy is enhanced in gefitinib-resistant cells and tissues

**Figure 3:**
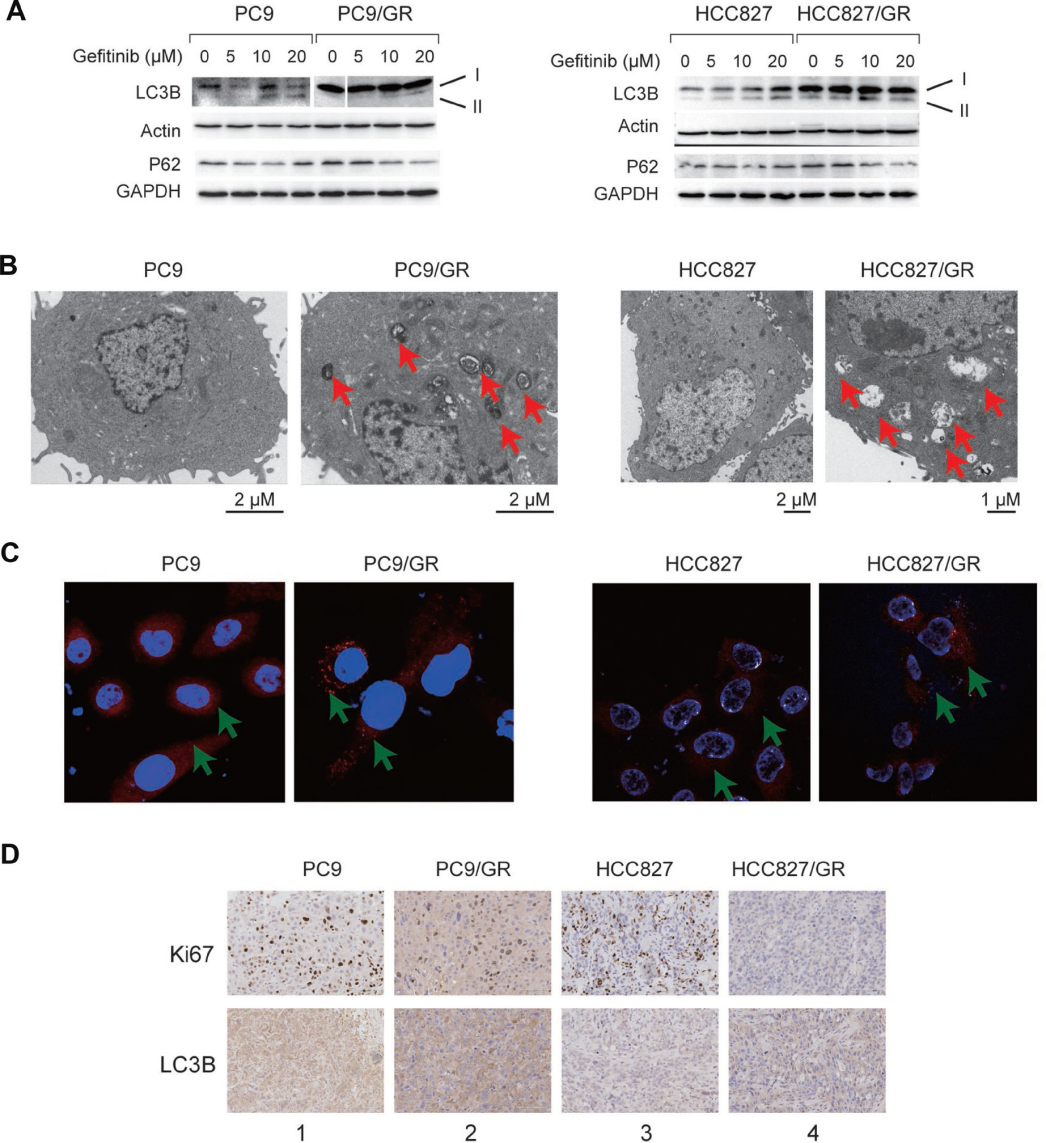
Autophagy is enhanced in the gefitinib-resistant NSCLC cells and tissues (**A**) WB detection of LC3B-I, LC3B-II, and P62 proteins in PC9 and PC9/GR cells (left panel) and HCC827 and HCC827/GR cells (right panel). Actin and GAPDH served as loading controls. (**B**) TEM images of PC9 and PC9/GR cells (left panel) and HCC827 and HCC827/GR cells (right panel). Red arrows point to autophagic vacuoles presented in gefitinib-resistant cells (PC9/GR and HCC827/GR), which are absent in gefitinib-sensitive cells (PC9 and HCC827). (**C**) Confocal microscopic images of the lysosomes in the PC9 and PC9/GR cells (left panel) and HCC827 and HCC827/GR cells (right panel). Red: lysosome tracker-stained lysosome. Blue: Hoechst 33258-stained nuclei. The green arrow points to the lysosome. (**D**) Immunohistochemical staining of Ki-67 and LC3B proteins in xenograft tumor tissues derived from PC9 (lane 1), PC9/GR (lane 2), HCC827 (lane 3), and HCC827/GR cells (lane 4).

Autophagy is enhanced in many tumor cells in response to drug treatment, which is normally associated with elevated lysosome activity [13–17]. To determine whether autophagy is also enhanced in the PC9/GR and HCC827/GR cells, we performed several experiments to detect autophagy and lysosome activity in these cells. First, we found that, LC3B-II, a marker for active autophagy, was up-regulated gradually upon the treatment with increasing amounts of gefitinib in PC9, PC9/GR, HCC827, and HCC827/GR cells (Figure 3A). However, p62 protein level was decreased gradually at the same time (Figure 3A); Second, using transmission electron microscopy (TEM), we found that the number of autophagic vacuoles, which are indicated by the red arrows, had increased dramatically in PC9/GR and HCC827/GR cells compared with PC9 and HCC827 cells (Figure 3B). We also observed increased numbers of autophagic vacuoles in the xenograft tumors derived from the resistant cells (Supplementary Figure 2). Third, we observed an increase in the formation of lysosome foci in the resistant cells, as detected by a fluorescent dye that specifically binds to the lysosomes, indicating an elevated level of lysosome activity (Figure 3C). Finally, we conducted an immunohistochemistry assay using the xenograft tumor tissues, and found that the expression level of Ki-67 (a cellular proliferation marker) was decreased, but the autophagy marker, LC3B, was increased in the drug-resistant cells (Figure 3D, comparing lane 1 vs. lane 2, or lane 3 vs. lane 4). These data reveal that autophagy and lysosomal activity were enhanced, but DNA replication was decreased, in the gefitinib-resistant cells, which is consistent with our mRNA-Seq analysis.

### Inhibition of autophagy suppresses gefitinib resistance

**Figure 4:**
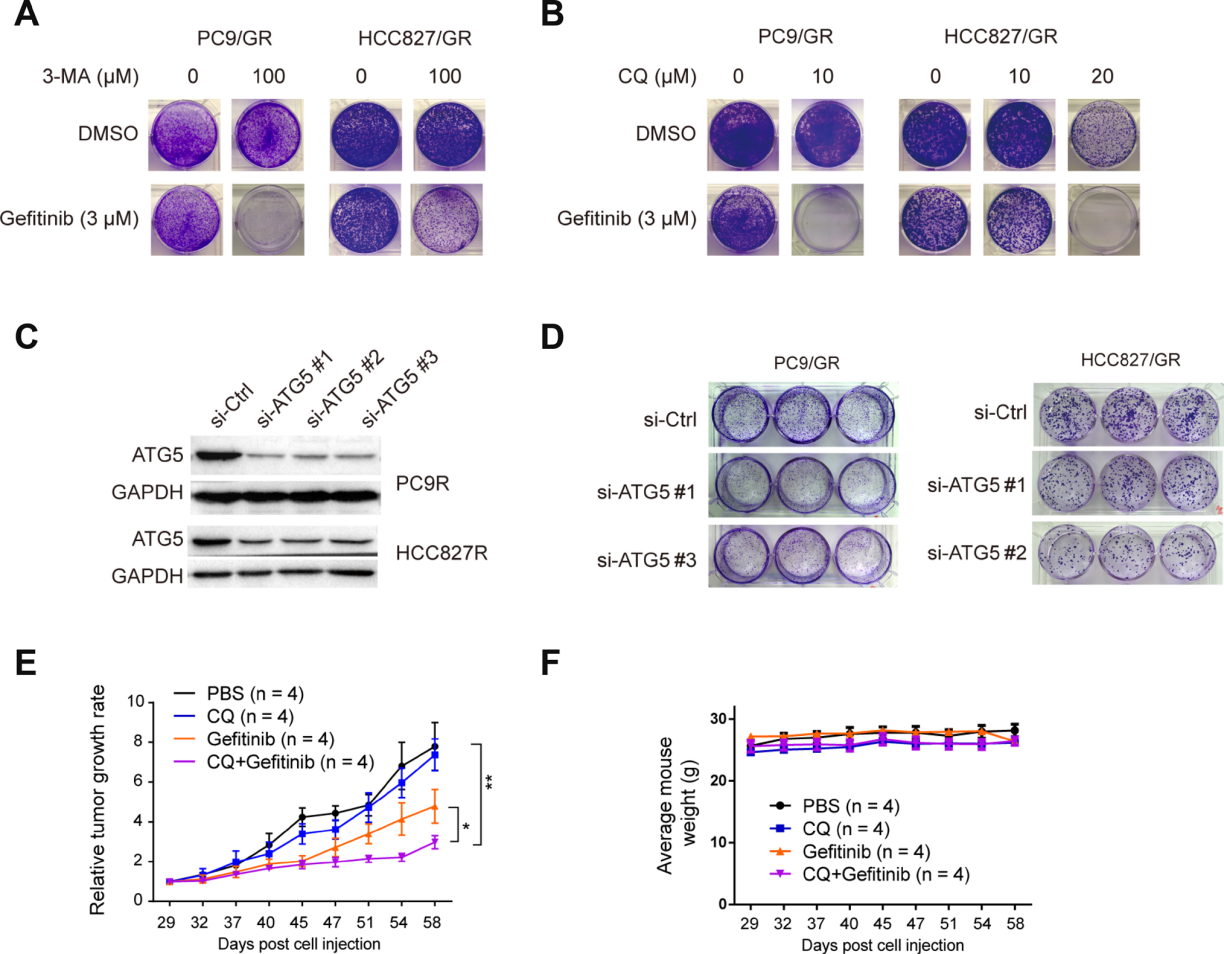
Inhibition of autophagy suppresses gefitinib resistance (**A**) PC9/GR and HCC827/GR cells were treated with either 0 or 100 µM 3-MA. (**B**) PC9/GR and HCC827/GR cells were treated with 0, 10, or 20 µM CQ for 2 days in combination with either DMSO (as the control) or 3 µM gefitinib, and then continued to proliferate and form colonies for 12 days. (**C**) WB validation of ATG5 knockdown by three different siRNA against ATG5 (si-ATG5#1, #2, or #3), compared with the control siRNA (si-Control) in PC9/GR and HCC827/GR cells. (**D**) Colony formation assay of PC9/GR and HCC827/GR cells transfected with si-ATG5#1, #2 or #3, compared with si-Control. (**E**) The relative tumor growth curves of PC9/GR derived xenograft tumors under different treatments. (**F**) Mice body weight curve over the same period of time as in (**E**).

To determine whether autophagy plays an important role in gefitinib resistance, we treated the gefitinib-resistant cells with two different autophagy inhibitors, 3-Methyladenine (3-MA), and Chloroquine (CQ). 3-MA inhibits autophagy by blocking autophagosome formation via the inhibition of class III PI-3 kinase [39]. CQ is a lysosomotropic agent that neutralizes the acidic pH of lysosomes, thereby preventing autophagic protein degradation and causing autophagosome accumulation [40]. Treatments with 3 µM gefitinib, 100 µM 3-MA, or 10 µM CQ alone could not suppress the colony formation capacity of PC9/GR and HCC827/GR cells, compared with cells treated with DMSO as the control. However, the combinatorial treatment of 3 µM gefitinib and 100 µM 3-MA or 20 µM CQ completely eradicated the colony formation capacity of PC9/GR and HCC827/GR cells (Figure 4A and 4B). This observation indicates that the autophagy inhibitors, such as 3-MA and CQ, work synergistically with gefitinib to kill gefitinib-resistant cells.

Next, to determine whether the ATG5 protein, which is an essential component in autophagosome formation, is necessary for gefitinib resistance, we knocked down the ATG5 expression in PC9/GR and HCC827/GR cells using three different siRNAs (Figure 4C), and found that the clonogenic outgrowth of PC9/GR and HCC827/GR cells transfected with si-ATG5 #1, #2, or #3 was significantly reduced compared with that of cells transfected with the control siRNA (Figure 4D). It is noteworthy that the effect caused by knocking down ATG5 was clearly weaker than those caused by the treatment of inhibitors (3-MA and CQ). This may be due to two reasons: 1) knocking down is the partial depletion of ATG5 gene expression, and 2) this knocking down effect by siRNA was transient, but not stable. Overall, our results suggest that the disruption of autophagy by ATG5 depletion can interfere with gefitinib resistance.

To determine the effect of autophagy inhibition on gefitinib-resistant tumor cell growth *in vivo*, we carried out xenograft experiments. Nude mice carrying PC9/GR cells derived xenograft tumors were divided into four groups, which received 100 µl of the following solutions by intragastrical injection twice per week: 1xPBS (as the control), 100 mg/kg gefitinib, 50 mg/kg CQ, and the mixture of gefitinib and CQ. Similar amounts of these drugs were used in other *in-vivo* studies [41, 42]. Relative tumor growth rate analysis showed that mice receiving the treatment of the mixture solution of gefitinib and CQ had the smallest relative tumor growth rate, as compared to the other three treatment groups (Figure 4E to 4F). Taken together, these observations indicate that autophagy inhibition can significantly suppress gefitinib resistant cell growth both *in vitro* and *in vivo*.

### ERK phosphorylation is activated in the gefitinib-resistant cells

**Figure 5:**
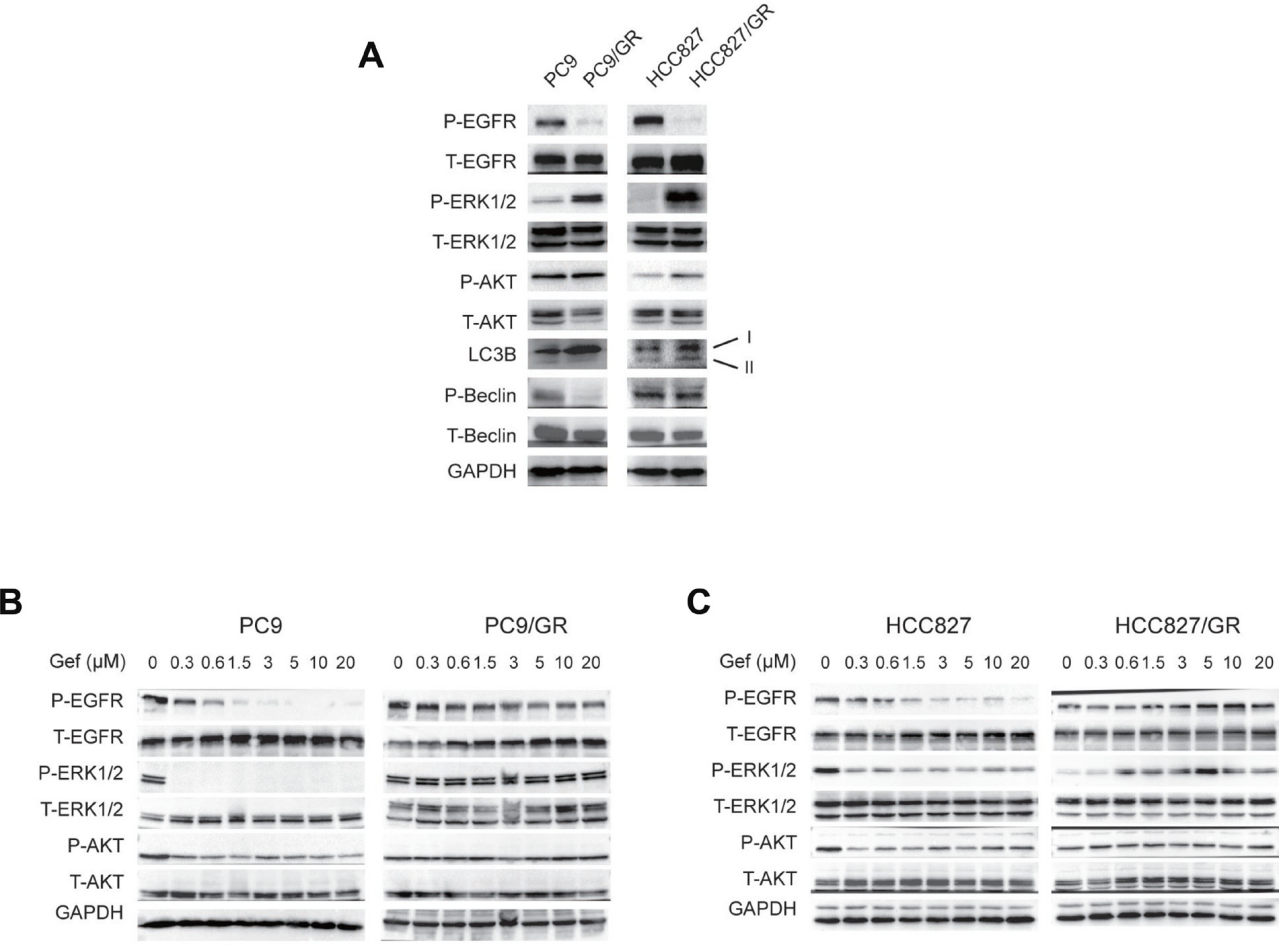
ERK phosphorylation is activated in gefitinib-resistant NSCLC cells (**A**) WB detection of the total (T-) and phosphorylated (P-) EGFR, ERK1/2, AKT, Beclin 1, and LC3B expression levels in PC9, PC9/GR, HCC827, and HCC827/GR cells. (**B**–**C**) WB detection of the total and phosphorylated EGFR, ERK1/2, and AKT levels in the PC9 and PC9/GR cells (B) and HCC827 and HCC827/GR cells (C) cultured in media containing 0, 0.3, 0.6, 1.5, 3, 5, 10, and 20 µM gefitinib for two days.

To study the signaling pathways involved in the gefitinib resistance, we performed WB to compare the activities of several signaling pathways between the sensitive cells without gefitinib treatment and the resistant cells in the presence of 3 µM gefitinib treatment. We found that EGFR (on Tyr1068) phosphorylation (P-EGFR) was significantly suppressed in PC9/GR and HCC827/GR cells in the presence of gefitinib (Figure 5A). However, ERK (on Thr202/Tyr204) and AKT (on Ser473) phosphorylation were enhanced in these cells, compared with their sensitive counterparts (Figure 5A). In addition, LC3B-II, which is associated with active autophagy, was increased in the gefitinib-resistant cells (Figure 5A). However, PC9/GR cells didn’t show LC3B-II expression as strong as those in HCC827/GR cells, this may due to the smaller gefitinib concentration (3 µM) used here (Figures 5A vs. 3A). Interestingly, Beclin 1 phosphorylation on Ser93 was inhibited in PC9/GR and HCC827/GR cells, which is consistent with a previous report that EGFR phosphorylation promotes Beclin 1 phosphorylation [43] (Figure 5A). These data suggest that EGFR phosphorylation is suppressed, but ERK and AKT signaling and autophagy are up-regulated in the gefitinib-resistant cells.

To examine the dynamic changes in the signaling pathways that respond to treatments with increasing gefitinib concentrations, we cultured these cells with increasing concentrations of gefitinib (0, 0.3, 0.6, 1.5, 3, 5, 10, and 20 µM) for 2 days, and found that ERK and AKT phosphorylation (P-ERK and P-AKT) rapidly decreased in PC9 and HCC827 cells in the presence of 0.3 µM or more gefitinib. However, P-ERK and P-AKT were stable and persisted in the PC9/GR and HCC827/GR cells (Figure 5B and 5C). Interestingly, while P-EGFR rapidly decreased in PC9, PC9/GR, and HCC827 cells under the treatment of increasing concentrations of gefitinib, P-EGFR decreased first, then increased later in HCC827/GR cells when treating with increasing concentrations of gefitinib, indicating the differential gefitinib response of PC9/GR and HCC827/GR cells (Figure 5B and 5C).

Finally, to determine the changes in the signaling events over time, we treated these cells with 3 µM gefitinib for various amounts of time (0, 0.5, 1, 2, 5, 12, 24, and 48 hours), and found that P-EGFR, P-AKT, and P-ERK were rapidly suppressed in PC9 cells at as early as 0.5 hour. However, in PC9/GR cells, P-EGFR remained at a constant low level, P-AKT and P-ERK increased at 5 hours after the initiation of the treatment (Supplementary Figure 3). Together, these results suggest that, although EGFR phosphorylation was suppressed, AKT and ERK phosphorylations were increased in gefitinib-resistant cells.

### Inhibition of ERK phosphorylation reverses gefitinib resistance by suppressing autophagy

**Figure 6:**
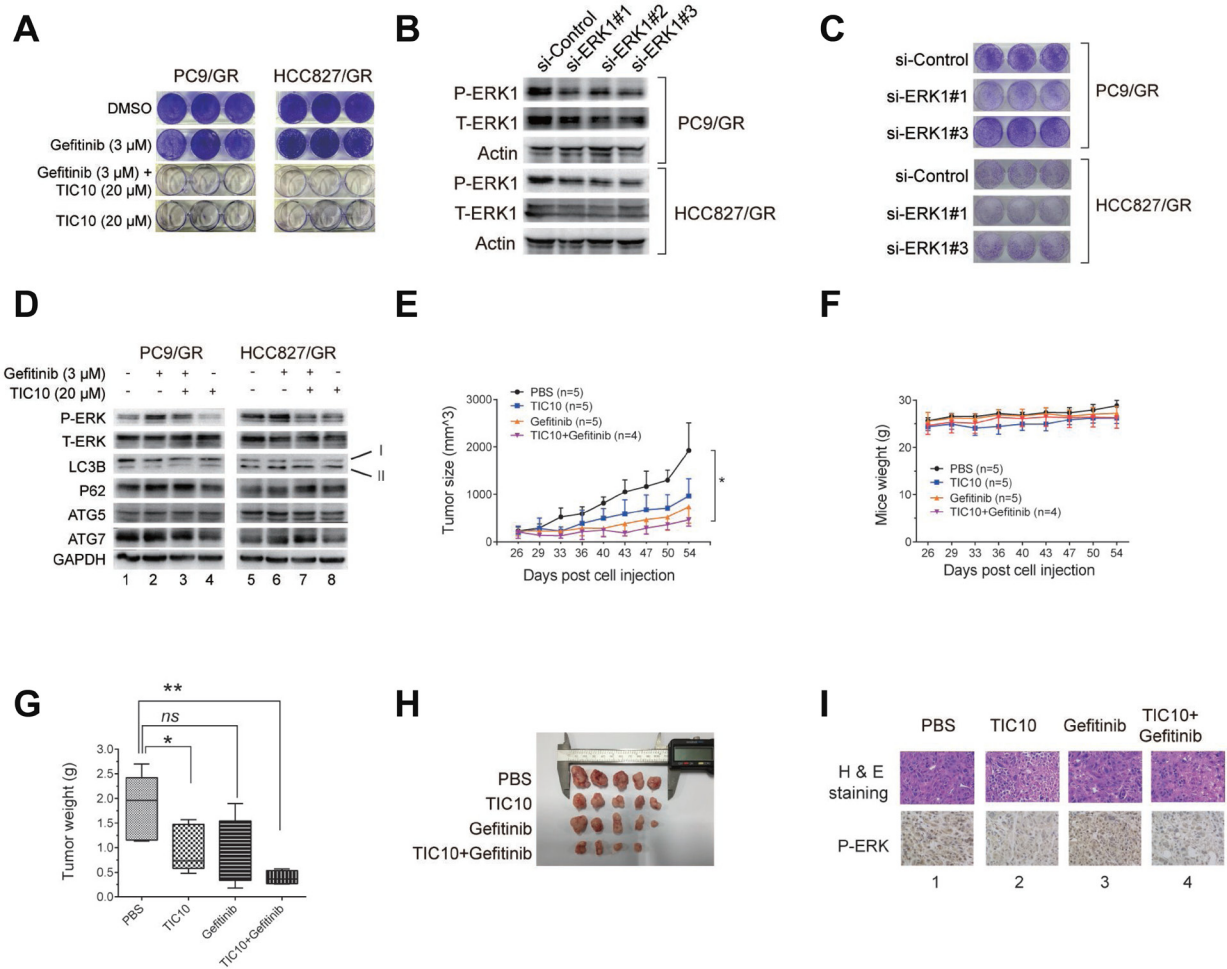
Inhibition of ERK phosphorylation reverses gefitinib resistance by suppressing autophagy (**A**) PC9/GR and HCC827/GR cells were cultured in media containing either DMSO (as the control), 20 µM TIC10 (ERK1/2 inhibitor), 3 µM gefitinib, or the combination of both TIC10 and gefitinib, for 12 days, followed by the colony formation assay. (**B**) WB validation of ERK1 knockdown by three different siRNAs against ERK1 (si-ERK1#1, #2, and #3) compared with the control siRNA (si-Control) in PC9/GR and HCC827/GR cells. (**C**) Colony formation assay of PC9/GR and HCC827/GR cells transfected with si-ERK1 #1, #2, or #3, compared with si-Control. (**D**) WB detection of the total and phosphorylated ERK1/2, LC3B, p62, ATG5, ATG7 levels in the PC9/GR and HCC827/GR cells cultured in the four treatment conditions described as above. (**E**–**F**) Growth curves of PC9/GR cells derived xenograft tumors (E) and mice body weights (F) in four treatment groups: PBS (as the control), gefitinib only, TIC10 only, and the combination of gefitinib and TIC10. (**G**) Images of isolated xenograft tumors from four treatment groups. (**H**) Comparison of average xenograft tumor weights from four treatment groups. (**I**) H&E staining and immunohistochemical images of phosphorylated ERK expression of xenograft tissues from four treatment groups.

To study whether ERK phosphorylation plays a key role in gefitinib resistance, we used a small molecule inhibitor of ERK1/2, TIC10, which was widely used in other studies [44, 45], and found that 3 µM gefitinib treatment alone did not affect the clonogenic outgrowth of the gefitinib-resistant cells compared with those treated with DMSO. However, 20 µM TIC10 alone significantly reduced the colony number of PC9R cells. Furthermore, the combination of 20 µM TIC10 and 3 µM gefitinib completely eradicated the colony number of the gefitinib-resistant cells (Figure 6A), indicating that the ERK signaling is necessary for gefitinib resistance and ERK inhibition combined with gefitinib abrogated gefitinib resistance.

Next, to determine whether the ERK signaling is necessary for gefitinib resistance, we knocked down the ERK1 expression in PC9/GR and HCC827/GR cells using three different siRNAs against ERK1 (Figure 6B), and found that the clonogenic outgrowth of PC9/GR and HCC827/GR cells transfected with si-ERK1 #1, or #3 was significantly reduced compared with the cells transfected with the siRNA control (Figure 6C). This result suggests that the disruption of ERK signaling by ERK1 depletion can interfere with gefitinib resistance.

To determine whether the effect of ERK inhibition on gefitinib resistance may be due to suppressed autophagy, we performed WB and found that, in the presence of 3 µM gefitinib, 20 µM TIC10 treatment decreased the expression of LC3B-II, but increased the expression of p62 in both cells (Figure 6D, comparing lane 2 vs. lane 3, or lane 6 vs. lane 7), indicating that autophagy was suppressed in the resistant cells as the result of ERK1/2 inhibition. In addition, there were no significant changes in ATG5 and ATG7 expressions in these cells (Figure 6D, comparing lane 2 vs. lane 3, or lane 6 vs. lane 7). These results suggest that TIC10 suppresses autophagy by inhibiting LC3B-II formation in gefitinib-resistant cells.

We further confirmed the above findings by using another ERK inhibitor, SCH772984. PC9/GR and HCC827/GR cells treated with both gefitinib and SCH772984 had significantly the fewer number of cell colonies, as compared to these cells treated with DMSO, gefitinib, or SCH772984 alone (Supplementary Figure 4A). In addition, WB analysis indicated that LC3B was down-regulated, while P62 was up-regulated in these cells treated with SCH772984, as compared to other treatments (Supplementary Figure 4B). Therefore, we concluded that ERK inhibition by SCH772984 can also suppress autophagy in these gefitinib-resistant cells.

To determine the effect of ERK1 inhibition on gefitinib-resistant tumor cell growth *in vivo*, we carried out xenograft tumor experiments. Nude mice carrying PC9/GR cells derived xenograft tumors were divided into four groups, which received 100 µl of the following treatments by intragastrical injection: 1xPBS (as the control), 100 mg/kg gefitinib, 25 mg/kg TIC10, and the combination of gefitinib and TIC10. Similar amounts of these drugs were used in other *in-vivo* studies [44, 45]. Comparisons of tumor growth curve and tumor weight showed that the mice receiving the combined treatment of gefitinib and TIC10 had the significantly slower tumor growth rate and the significantly smaller tumor weight than those receiving the control treatment, while the average mice weight is comparable among all four treatment groups (Figures 6E to 6H). Interestingly, TIC10 treatment alone also reduced the tumor size due to its antitumor effect, but its inhibitory effect was not strong as the combined treatment of gefitinib and TIC10. Subsequent immunochemical staining images showed that the ERK phosphorylation level was reduced in xenograft tumor tissues receiving TIC10 treatment (Figure 6I, lanes 2 and 4), compared to those without TIC10 treatment (Figure 6I, lanes 1 and 3).

Therefore, we concluded that pharmacological inhibition of the ERK signaling can significantly reduce gefitinib-resistant tumor cell growth both *in vitro* and *in vivo* by suppressing autophagy.

## DISCUSSION

In this study, we generated two novel gefitinib-resistant NSCLC cell lines and compared their IC_50_ of gefitinib, anti-apoptosis abilities, and growth rates with gefitinib-sensitive NSCLC cells. In addition, we found that multiple pathways are dysregulated in gefitinib-resistant cells. Importantly, autophagy was enhanced in the gefitinib-resistant cells, and the inhibition of autophagy suppressed the gefitinib resistance. We also found that phosphorylations of ERK and AKT are activated in resistant cells. Inhibition of ERK phosphorylation greatly suppressed gefitinib resistance both *in vitro* and *in vivo*. Based on these observations, we propose a model for the mechanism of gefitinib resistance (Supplementary Figure 5). In this model, ERK phosphorylation promotes autophagy by stimulating LC3B-II formation, and p62 degradation. This leads to gefitinib resistance in NSCLC cells.

Multiple signaling pathways have been found to be important for drug resistance. For example, hyperactive ERK and persistent mTOR signaling promote vemurafenib resistance in papillary thyroid cancer cells [46]; mTOR was associated with drug resistance in lung adenocarcinoma after radiation combined with TKI, and mTOR inhibition reverses drug resistance in lung adenocarcinoma after combined radiation and TKI therapy [47]; dual inhibition of AKT signaling/FLT3-ITD by the well-established orally available AKT inhibitor, A674563, overcomes FLT3 ligand-induced drug resistance in FLT3-ITD positive AML [48].

Our mRNA-Seq-based transcriptome analysis reveals several significantly up- or down-regulated pathways that are associated with gefitinib resistance. In addition to enhanced lysosome activity, other cellular pathways, such as ECM-receptor interaction, cell adhesion molecules (CAMs), and the O-glycan biosynthesis pathways, may also be involved in regulating gefitinib resistance and autophagy. For example, the ECM protects small-cell lung cancer cells from DNA damage-induced cell cycle arrest and apoptosis through beta1 integrin-dependent activation of PI3 kinase [49]. Integrin-mediated adhesion regulates ERK nuclear translocation and phosphorylation of Elk-1 [50]. We are currently studying how these pathways affect gefitinib resistance, which may reveal new information on the molecular mechanism of acquired gefitinib resistance.

In our study, we found that inhibition of autophagy by CQ or inhibition of ERK by TIC10 can suppress the gefitinib resistance *in vivo*. In fact, many clinical trials are currently underway to investigate the effect of autophagy inhibitors (CQ and hydroxychloroquine (HCQ)) or the ERK inhibitor (TIC10, ONC201 (other name)) in treating advanced solid tumors. For example, along with other therapies, CQ and HCQ are being tested in clinical trials to treat cancer [13]**.** The NCT02378532 and NCT02333890 clinical trials to treat glioblastoma and breast cancer are currently evaluating the effect of CQ on radioresistant cancers. In addition, clinical studies of oral ONC201 are underway in patients with relapsed non-hodgkin’s lymphoma, adult recurrent glioblastoma, and relapsed acute leukemia, etc. Even though our data reveal the importance of the ERK signaling in gefitinib-resistant NSCLC cells, there are still many unanswered questions left: how the ERK signaling is enhanced during the gefitinib resistance, and how it promotes autophagy. Further studies are needed to address these questions.

Overall, our study uncovers new cellular pathways involved in gefitinib resistance, and reveals that the ERK signaling plays an important role in promoting autophagy-induced gefitinib resistance. Our work also suggests that inhibitors targeting the ERK signaling may be effective in preventing acquired gefitinib resistance in NSCLC patients.

## MATERIALS AND METHODS

### Ethics

All animal experiments were performed using male BALB/C nude mice (4–5 weeks old). The mice were purchased from the SLAC Laboratory Animal Center (Shanghai, China) and cared for in accordance with the Guide for the Care and Use of Laboratory Animals from the National Institutes of Health. All animal experimental protocols performed in this study were approved by the Institutional Animal Care and Use Committee at Tongji University (IACUC No. 1201).

### Cell culture

Four human NSCLC cell lines were used in this study, including two gefitinib-sensitive cell lines, PC9 and HCC827, and two gefitinib-resistant cell lines, PC9/GR and HCC827/GR. PC9 and PC9/GR cells were grown in DMEM medium (Hyclone, GE Healthcare Life Sciences, Utah, USA). HCC827 and HCC827/GR cells were grown in RPMI-1640 (Hyclone). All medium contained 10% FBS (Gibco) supplemented with penicillin (100 U/ml) and streptomycin (100 mg/ml) (Life Technologies). In addition, gefitinib was added to the medium of PC9/GR and HCC827/GR cells at a final concentration of 3 µM. Cells were incubated at 37° C in a humidified atmosphere with 5% CO_2_.

### Colony formation assay

Colony formation assays were performed as previously described [51].

### Cell proliferation assay

Colony proliferation assays were performed as previously described [51].

### siRNA transfection

The ATG5 and ERK1 siRNAs were synthesized by GenePharma Inc. (Shanghai, China). The transfections were performed with Lipofectamine 2000 (11668019, Invitrogen, CA, USA) according to the manufacturer’s protocol. The total RNA or cell lysates were prepared 48 h after transfection and were used for Western blotting analyses, respectively.

The sequences for the siRNAs against ATG5 are as follows: #1: sense (5'-3') GACGUUGGUAACUGACAAATT and antisense (5'-3') UUUGUCAGUUACCAACGUCTT; #2: sense (5'-3') GUCCAUCUAAGGAUGCAAUTT and antisense (5'-3') AUUGCAUCCUUAGAUGGACTT; #3: sense (5'-3') GACCUUUCAUUCAGAAGCUTT and antisense (5'-3') AGCUUCUGAAUGAAAGGUCTT.

The sequences for the siRNAs against ERK1 are as follows: #1: sense (5'-3') CCUUCGAACAUCAGACCUATT and antisense (5'-3') UAGGUCUGAUGUUCGAAGGTT; #2: sense (5'-3') GAGAUGUCUACAUUGUGCATT and antisense (5'-3') UGCACAAUGUAGACAUCUCTT; #3: sense (5'-3') CUGCGACCUUAAGAUUUGUTT and antisense (5'-3') ACAAAUCUUAAGGUCGCAGTT.

### Western blotting (WB)

Western blotting was performed as previously described [51]. The antibodies and catalogue numbers are listed (Supplementary Table 1).

### RNA isolation and real-time RT-PCR

Total RNA extraction from cells and real-time RT-PCR were performed as previously described [51]. The PCR primer names and sequences are listed (Supplementary Table 2).

### IC_50_ determination

Totals of 2,000–4,000 PC9 and HCC827 cells or 3,000 PC9/GR and HCC827/GR cells were seeded in 96-well plates, incubated with the indicated concentrations of gefitinib for three or four days, and assessed for the cell survival with the MTS reagent (Promega G3580). Survival data were analyzed, and IC_50_ was determined using the Prism GraphPad software (version 6.01).

### mRNA-seq library preparation

The total RNA was extracted from the PC9 and PC9/GR cells using RNAprep Pure (Tiangen, DP430, Shanghai, China), and the mRNAs were purified using the Dynabeads mRNA purification kit (Invitrogen, 61006). The cDNAs were synthesized and used to construct a library with the NEBNext Ultra RNA Library Prep Kit (NEB, E7530). The libraries were sequenced on the Illumina HiSeq2000 platform via a 1x50bp single-end sequencing at BGI Tech Solutions Co., Ltd. (Shenzhen, China).

### RNA-seq data analysis

The RNA-Seq reads were aligned to the hg19 genome assembly using TopHat (version 1.1.4) with the default parameters [52]. The expression index was generated using GFOLD V1.1.3 job count [30] from the bam files from TopHat. The differentially expressed genes were ranked using GFOLD V1.1.3 job diff [30]. The final output file contains all genes with a GFOLD value, which could be considered as the reliable log2-fold change between the control (PC9) and treatment (PC9/GR) conditions. Pathway enrichment analyses were performed by DAVID (http://david.abcc.ncifcrf.gov) [53]. The heatmap clustering analysis was done using K-means clustering method in R (version 3.2.3).

All sequencing data were deposited in the NCBI Gene Expression Omnibus (GEO) (http://www.ncbi.nlm.nih.gov/geo/) under accession number GSE74253.

### Small molecule inhibitors

The following small molecule inhibitors were used in this study: gefitinib, an EGFR inhibitor (IRESSA, AstraZeneca); 3-Methyladenine (3-MA) (Selleck, S2767); Chloroquine Phosphate (CQ) (Selleck, S4157); and TIC10, an ERK and AKT inhibitor (MCE, HY-15615A).

### Transmission electron microscopy (TEM)

A 1 cm^3^ block of tissue or 1 × 10^6^ cells were fixed in an electron microscope fixing solution (Wuhan Google Biotechnology, #G1102), and sent for TEM imaging analysis using a Tecnai G2 200kV Transmission Electron Microscope (FEI, USA), with photos taken at various magnifications.

### Fluorescent confocal microscopy

The cells cultured in 12-well plates were fixed in 4% Paraformaldehyde, washed with PBS, and stained with 500 µl of Lyso-tracker Red (Beyotime Biotechnology, #C1046) and Hoechst 33258 (Beyotime Biotechnology, #C1011). An anti-fluorescent quenching liquid (Beyotime Biotechnology #P0126) was added to the solution. The cells were observed using a 90i confocal microscope (Nikon, Japan).

### Immunohistochemistry

The immunohistochemistry experiments were performed by Wuhan google technology Co. LTD. (http://servicebio.cn/, Wuhan, China). Briefly, the formalin-embedded tumor tissue samples were sliced and subjected to immunohistochemistry using the following primary antibodies: Ki67 (ab15580, Abcam, USA), LC3B (PM036, MBL, USA). The primary antibodies were detected with EnVision™ Detection Systems Peroxidase/DAB, Rabbit/Mouse (K5007, Dako, USA).

### Apoptosis assay

Apoptosis assays were performed as previously described [54].

### Xenograft assay

Male nude mice were purchased from Shanghai SLAC Laboratory Animal Center. A total of 2 × 10^6^ PC9/GR cells suspended in 100 μL 1xPBS was injected subcutaneously into the right axillary part of 5-week old mice. When the tumor size reached 100 mm^3^, 20 mice were randomly divided into four groups. Mice in different groups were intragastrically injected with 100 µl of the following solution containing: 1xPBS, CQ (50 mg/kg), gefitinib (100 mg/kg), CQ followed by gefitinib, respectively. Relative tumor growth rate was calculated as the ratio of the tumor size at a given time point vs. the tumor size at the time point of drug injection the first time.

For TIC10 *in vivo* assay, PC9/GR cells derived xenograft tumors were allowed to grow to around 100 mm^3^, then mice were divided into four groups. Mice in different groups were intragastrically injected with 100 µl of the following solution containing: 1xPBS, TIC10 (25 mg/kg), gefitinib (100 mg/kg), the mixture of TIC10 and gefitinib, respectively.

Treatment was done twice per week. Tumor size was measured every week using the digital caliper, and its volume (v) was calculated based on this formula: v = 0.5 ^*^ a ^*^ b2, with a = long diameter, b = short diameter. After eight measurements, tumor tissues were isolated, photographed, and weighted. All procedures performed in this study are approved by the Institutional Animal Care and Use Committee at Tongji University.

### Statistical analysis

Student’s *T* test was used to determine the significance of the differences between two groups. *P* < 0.05 is considered to be significant. ^*^P < 0.05, ^**^P < 0.01, ^***^P < 0.001, ^****^P < 0.0001.

## SUPPLEMENTARY MATERIALS FIGURES AND TABLES






