# Supplementary material for: ERK inhibition represses gefitinib resistance in non-small cell lung cancer cells
Source: Oncotarget. 2018 Jan 10;9(15):12020–34. doi: 10.18632/oncotarget.24147 (PMC5844726; doi:10.18632/oncotarget.24147)
Supplement: Supplementary file 1 [file oncotarget-09-12020-s001.pdf]

ERK inhibition represses gefitinib resistance in non-small cell lung cancer cells

SUPPLEMENTARY MATERIALS

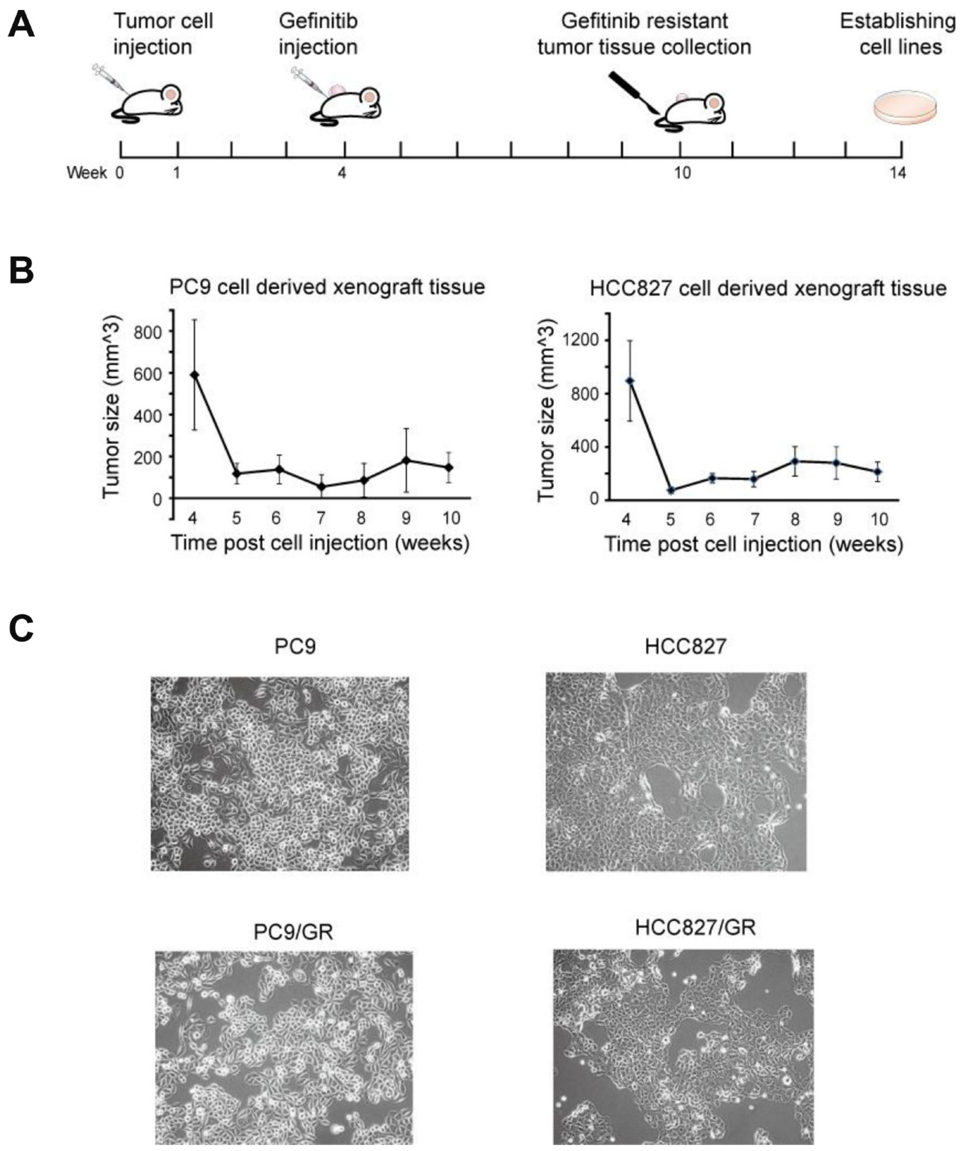

Supplementary Figure 1: ***In vivo*** **screening of gefitinib-resistant PC9/GR and HCC827/GR NSCLC cells.** (**A**) Schematic of the experimental workflow for isolating the gefitinib-resistant PC9/GR and HCC827/GR cells from the xenograft tumor tissues. (**B**) The tumor size was measured every week from weeks 4 to 10. (**C**) Microscopic images of PC9, PC9/GR, HCC827, and HCC827/GR cells.

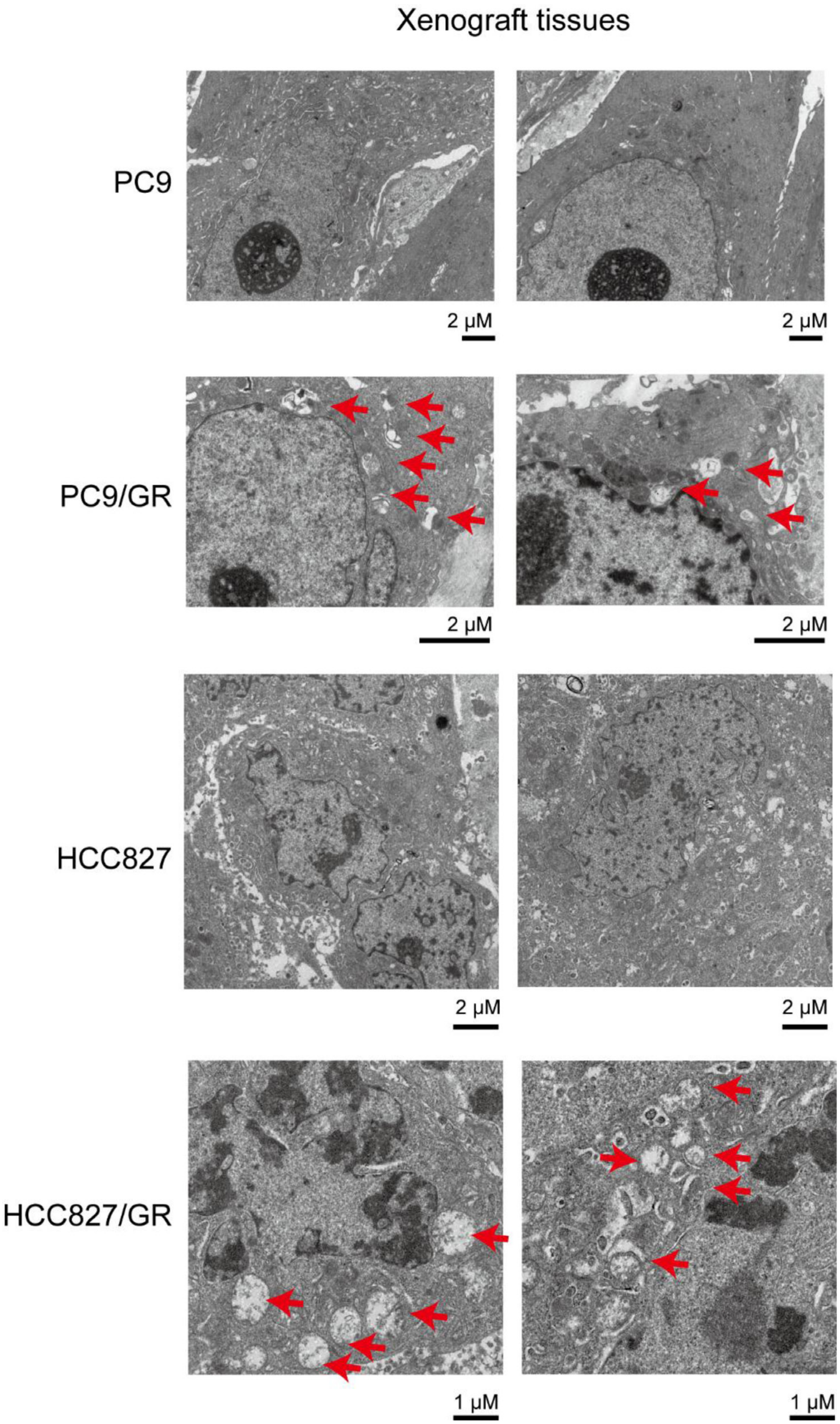

Supplementary Figure 2: TEM images of the xenograft tissues derived from PC9, PC9/GR, HCC827, and HCC827/GR cells. The xenograft tissues derived from the following cells: PC9, PC9/GR, HCC827, and HCC827/GR (from top to bottom) were dissected, and sent for TEM imaging analysis. The autophagic vacuoles are pointed by the red arrows in PC9/GR and HCC827/GR cells, while they are absent in PC9 and HCC827 cells.

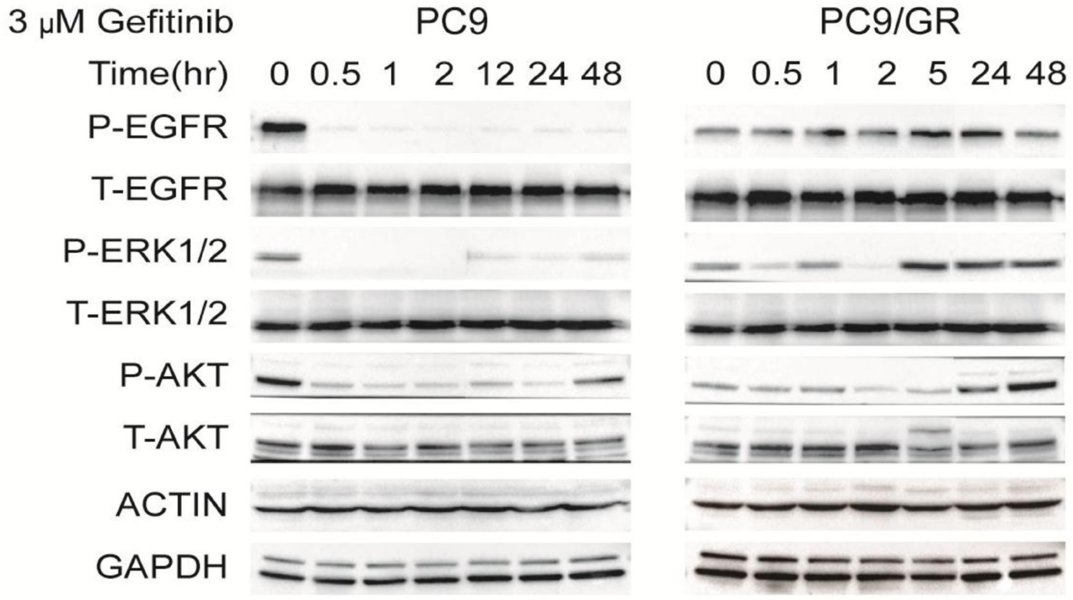

Supplementary Figure 3: WB detection of changes in signaling pathways over time in PC9 and PC9/GR cells. WB detection of the total (T-) and phosphorylated (P-) EGFR, ERK1/2, and AKT protein levels in PC9 cells (left) and PC9/GR cells (right) cultured in media containing 3 μM gefitinib for various lengths of time (0, 0.5, 1, 2, 5, 12, 24, and 48 hours). Actin or GAPDH serves as the loading control.

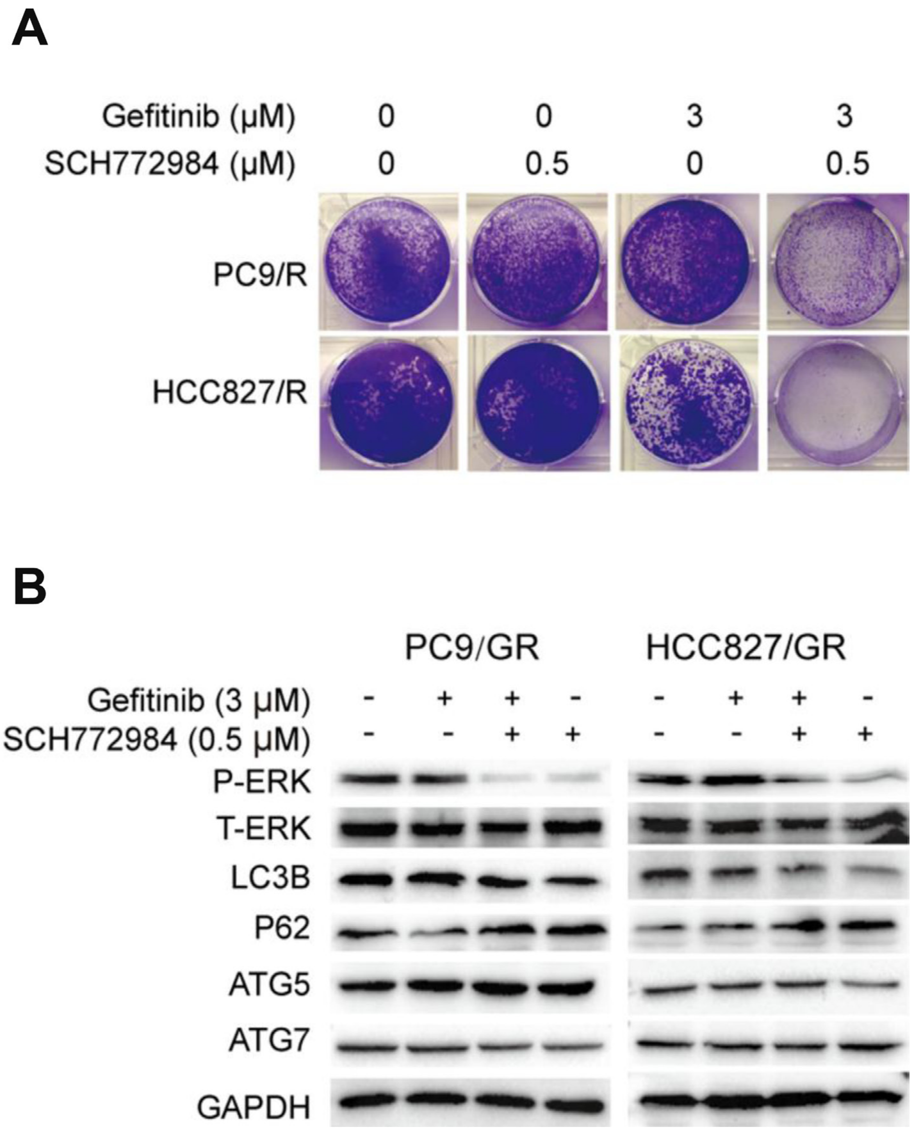

Supplementary Figure 4: The effect of ERK1/2 inhibition by SCH772984 on gefitinib resistance and autophagy in PC9R and HCC827R cells. (**A**) PC9R cells (top) and HCC827R cells (bottom) were cultured in media containing either DMSO (Control), 0.5 μM SCH772984 (ERK1/2 inhibitor), 3 μM gefitinib, or the combination of both SCH772984 and gefitinib, and cultured for additional 12 days followed by colony formation assays; (**B**) WB detection of the total and phosphorylated ERK1/2, LC3B, p62, ATG5, ATG7, Beclin 1 levels in the PC9R and HCC827R cells cultured in the four treatment conditions described above.

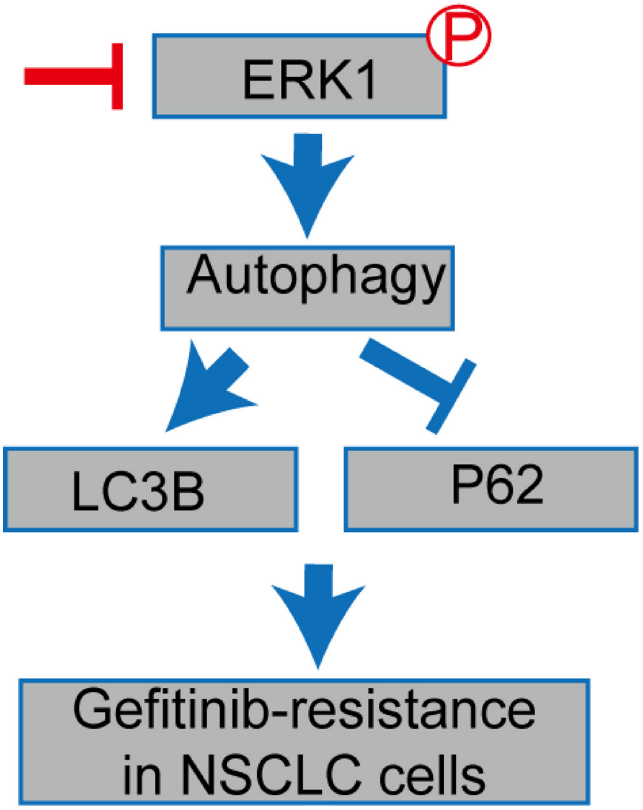

Supplementary Figure 5: The model of ERK-mediated autophagy and gefitinib resistance. Activated ERK1/2 enhances autophagy, in which LC3B-II is up-regulated and p62 is down-regulated. This leads to gefitinib-resistant NSCLC cells. Inhibition of the ERK signaling results in the suppression of gefitinib resistance in NSCLC cells.

Supplementary Table 1: Antibody names and catalogue numbers

Antibody name	Catalogue number
LC3B	CST #3868
GAPDH	Sigma G9545
Actin	CST #4970
ATG7	CST #8558
ATG5	CST #2630
P-EGFR (Tyr1068)	CST #3777
EGFR	CST #4267
P-ERK (Thr202/Tyr204)	CST #4370
ERK	Santa Cruz Biotechnology: sc-94
P-AKT (Ser473)	CST #4060
AKT	Santa Cruz Biotechnology: sc-1619
p62	CST #8025
P-Beclin 1 (Ser93)	CST #14717
Beclin 1	CST #3495
Goat anti-rabbit IgG	CST, #7074S
Goat polyclonal anti-mouse IgG	Abcam, #ab136815

Supplementary Table 2: PCR primer names and sequences

PCR primer name	sequence
CANX forward	5′-AACCCGAAGGCTGGTTAGAT-3′
CANX reverse	5′-CATCCAGGAGCTGACTCACA-3′
EDEM1 forward	5′-AAGCCTGCAATGAAGGAGAA-3′
EDEM1 reverse	5′-CTATCAGCACCTGCAGTCCA-3′
RB1CC1 forward	5′-GCGGAACAACAGACCAATTT-3′
RB1CC1 reverse	5′-CGTTCTTGTTGCTGCATTGT-3′
FOXO1 forward	5′-CTGCATCCATGGACAACAAC-3′
FOXO1 reverse	5′-AGGCCATTTGGAAAACTGTG-3′
MAPK1 forward	5′-GTTGGTACAGGGCTCCAGAA-3′
MAPK1 reverse	5′-TGGAAAGATGGGCCTGTTAG-3′
HSPB8 forward	5′-GCTTCAAGCCAGAGGAGTTG-3′
HSPB8 reverse	5′-TGCAGGAAGCTGGATTTTCT-3′
CDKN1A forward	5′-GGAAGACCATGTGGACCTGT-3′
CDKN1A reverse	5′-GGCGTTTGGAGTGGTAGAAA-3′
ATG16L2 forward	5′-TGACAGCTGCCAAATTCAAG-3′
ATG16L2 reverse	5′-GGACATTGATGGTCCTGGAG-3′
GAPDH forward	5′-CCGGGAAACTGTGGCGTGATGG-3′
GAPDH reverse	5′-AGGTGGAGGAGTGGGTGTCGCTGTT-3′

Supplementary Table 3: The GFOLD output files of the mRNA-Seq data (PC9/GR compared to PC9 cells). Two biological replicates were used for mRNA-Seq experiments, which are labeled as "PC9GRvsPC9" and "PC9GRvsPC9_rep" in the table. See Supplementary_Table_3

Supplementary Table 4: The KEGG pathway enrichment analysis of top 2000 up or down-regulated genes in PC9/GR cells, as compared with those in PC9 cells. See Supplementary_Table_4
